# Effects of Sensor Location on Dynamic Load Estimation in Weigh-in-Motion System

**DOI:** 10.3390/s18093044

**Published:** 2018-09-12

**Authors:** Tianhao Qin, Mengxiang Lin, Ming Cao, Kaiya Fu, Rong Ding

**Affiliations:** State Key Laboratory of Software Development Environment, School of Mechanical Engineering & Automation, Beihang University, Beijing 100083, China; zy1607505@buaa.edu.cn (T.Q.); linmx@buaa.edu.cn (M.L.); caoming123@buaa.edu.cn (M.C.); sy1607504@buaa.edu.cn (K.F.)

**Keywords:** weigh-in-motion, embedded sensor network, sensor layout

## Abstract

In recent years, weigh-in-motion systems based on embedded sensor networks have received a lot of attention. However, how to improve the accuracy of multi-sensor weigh-in-motion (WIM) systems while keeping costs low remains a challenge. In this paper, a numerical simulation method is presented to analyze the relationship between sensor location and the accuracy of static weight estimation. The finite element model of a WIM system is developed, which consists of three parts: a pavement model, a moving load model and two types of sensor models. Analysis of simulation results shows that the ability of sensing dynamic load is closely related to the installation depth of sensors and pavement material. Moreover, the distance between the moving wheel and sensors has a great impact on estimating performance. Gaussian curve fitting could be used to reduce weighing error within a limited range. Our work suggests that much more attention should be paid to the design of the sensor layout of a WIM system.

## 1. Introduction

The goal of a weigh-in-motion system (WIM) is to measure the static weights of vehicles travelling at highway speeds [1,2]. However, the accuracy of WIM is limited by a variety of factors. As a solution to the problem, the multiple sensor WIM has been proposed, in which an array of sensors is deployed along the pavement and the responses of pavements are collected [3,4].

Due to the road surface unevenness, the dynamic characteristics of vehicles and complex interaction process between vehicles and pavements, the measurement of WIM sensors is instantaneous load varying with a certain frequency. Many research efforts have been devoted to accurately estimate static weight based on dynamic load [5]. The arrangement of rows of sensors in the driving direction has been studied theoretically and experimentally in order to cover the full dynamic response period. Furthermore, various estimation algorithms have been proposed so as to eliminate the errors caused by the dynamics of vehicles as many as possible [6,7]. 

Different from the above work, our study focuses on the relationship between the measured dynamic loads and sensor locations. As indicated by previous studies, the dynamic force applied to the ground by a moving wheel is approximatively distributed following a Gaussian distribution [4,8]. In the ideal case, that is, the sensor is just below the tire, the output of the sensor is the mean of the Gaussian distribution, which is usually used to estimate dynamic load. However, the track of a travelling vehicle on the road is unpredictable in practice. How to deploy sensors to measure the exact values of dynamic loads is important but still not valued.

In this paper, a numerical simulation method is presented to analyze the effects of sensor location on accuracy of dynamic load estimation. The finite element model of a WIM system is built, which consists of three parts: a pavement model, a moving load model and two types of sensor models. The model allows us to install any number of sensors at any given place under the pavement. Dynamic simulation of embedded sensors subjected to a moving load provides more accurate insight into the characteristics of responses, which will obviously benefit the design of the sensor layout of a WIM system.

The rest of this paper is organized as follows. In Section 2, we discuss the related work. Section 3 describes the dynamic load model of the vehicle and the pavement response model under a moving load. Section 4 builds the finite element model of pavement and sensor under the whole vehicle moving load. Section 5 analyzes weighing errors in the case of the different depth of sensors and lateral offsets of wheels and sensors. We conclude in Section 6. 

## 2. Related Work 

WIM systems vary from sensor to senor. The early WIM systems use a single large and expensive sensor, such as capacitive mats and bending plates. Recently, the array of sensors is introduced, in which sensors such as strain gauges and optic fibers are embedded to collect the responses of pavements. We focus on the latter in this paper. The closely related work is reviewed in the following.

An experimental investigation with a multi-sensor WIM was conducted to measure the dynamic response of concrete pavement under the travelling vehicles [9]. The system consists of a sensor network with 120 sensors, including two types of strain gages (ESG), linear displacement transducers (LDT), vertical accelerometers and thermocouples. Two rows of ESGs are embedded at different depths of the pavement, four rows of ESGs are deployed perpendicularly to the direction of travel and 15 rows of ESGs are arranged in the direction of travel. In their following work, a finite element model is built and the data measured by the sensor network is used to correct the finite element model [10]. Both finite element analysis and field tests show that embedded multi-sensors detect dynamic response well.

A novel WIM system based on embedded strain sensors was proposed [3]. There is a total of five strain sensors and only three sensors located in the center of the road are used to measure axle load. This study indicates that it is feasible to measure the axle weight with embedded strain sensors. Moreover, the mean approach by sampling multiple sensors gives better performance. Recently, optimization of WIM systems based on embedded sensors was performed [11]. The optimization framework establishes the relationship between the sensor signal and the measurement dynamic range.

An integrated traffic monitoring system was developed to monitor road damage status, vehicle speed, vehicle weight and traffic volume [4]. A variety of embedded sensors including CTL asphalt strain gauges, vertical strain gauges, load cells and a moisture sensor are used. Strain sensors are divided into two groups and are symmetrically deployed along the middle of the road. Each sensor group contains seven horizontal strain gauges and two vertical strain gauges. The lateral placement of horizontal sensors is chosen for more chance for the sensors to be passed.

The effect of inflation pressure for a specific tire configuration on pavement responses and distress development was investigated [12]. Two kinds of strain gauges are used, including rosettes strain gauge and normal strain sensor. Two rows of sensors are buried in different depth under the pavement and one row of sensors is arranged in the direction of travel and one is at the direction perpendicular to the direction of travel. The numerical simulation was also conducted and the measured strain responses were compared to the simulated ones.

An in-pavement 3-D glass fiber-reinforced polymer packaged fiber Bragg grating sensor (3-D GFRP-FBG) was developed to classify vehicles [13,14]. The 3D GFRP-FBG sensor consists of three components in different directions so as to detect vertical strain, longitudinal strain and transverse strain, respectively. The sensor network consists of two 3D GFRP-FBG sensors installed under the wheel path to be more accurate for vehicle passing measurements. Further research shows that this sensor network can also be used to form a dynamic weighing system to measure vehicle weight [15].

## 3. Model of Vehicle and Pavement

In this section, the vehicle dynamic load model and pavement motion model are created, which serve as the theoretical basis to develop finite element simulation in following section. Specifically, the dynamic tire force equation is derived from a quarter vehicle model and the model of road roughness. The generalized Maxwell model is introduced as the viscoelastic model of asphalt pavements. Furthermore, combining with the finite element theory, the motion model of pavement is obtained.

### 3.1. Vehicle Dynamic Load Model

**Figure 1:**
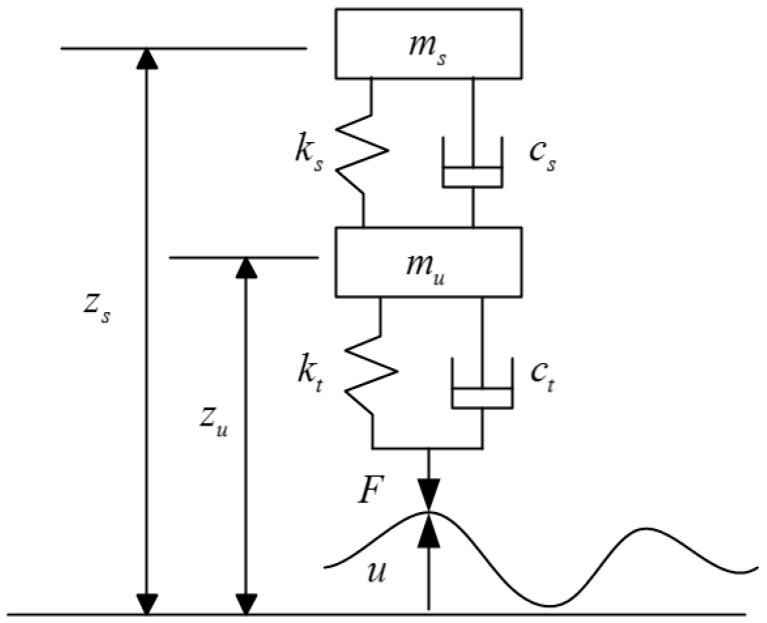
Quarter vehicle model with two-degree-freedom. $m_{s}$ is sprung mass (including vehicle body mass), $m_{u}$ is unsprung mass (including tire, rim and axle mass), $k_{s}$,$c_{s}$ are suspension stiffness and damping, $k_{t}$,$c_{t}$ are tire stiffness and damping, $z_{s}$,$z_{u}$ are displacement of $m_{s}$,$m_{u}$ from static balance position, u is road roughness.

The vehicle model is an essential part in the study of vehicle–road interaction. The quarter vehicle model has been widely used in various research works because it has sufficient frequency content for predicting dynamic loads [16,17]. As shown in Figure 1, the quarter vehicle model is represented by a vehicle body mass and a tire mass connected with a spring and dashpot [18].

The kinematics equation for the quarter vehicle model is as follows:

(1) $$M\ddot{q}+C\dot{q}+Kq=p$$

where M, C and K are mass matrix, damping matrix and stiffness matrix respectively, q and p are system vertical displacement and external force. The definition of the variables in the above equation is the same as in the literature [19]:

$$q=\left[\begin{array}{c} z_{s}\\ z_{u} \end{array}\right],\;p=\left[\begin{array}{c} 0\\ c_{t}\dot{u}+k_{t}u \end{array}\right],\;M=\left[\begin{array}{cc} m_{s} & 0\\ 0 & m_{u} \end{array}\right],\;C=\left[\begin{array}{cc} c_{s} & -c_{s}\\ -c_{s} & c_{s}+c_{t} \end{array}\right],\;K=\left[\begin{array}{cc} k_{s} & -k_{s}\\ -k_{s} & k_{s}+k_{t} \end{array}\right]$$

Due to road roughness u, the moving vehicle generates a dynamic load, also referred to as dynamic tire force, which can be expressed as Equation (2). The interested reader is referred to the literature for derivation details [19].

(2) $$F_{d}=c_{t}\left(\dot{z_{u}}-\dot{u}\right)+k_{t}\left(z_{u}-u\right)$$

The vehicle static weight is indicated in Equation (3). Finally, the total tire force consisting of the dynamic tire force and vehicle static weight can be written as Equation (4):

(3) $$F_{s}=\left(m_{s}+m_{u}\right)g$$

(4) $$F=c_{t}\left(\dot{z_{u}}-\dot{u}\right)+k_{t}\left(z_{u}-u\right)+\left(m_{s}+m_{u}\right)g$$

Road roughness *u* is obtained by a road surface model in the form of its power spectral density (PSD) [19], which is defined as:

(5) $$S_{q}\left(\Omega \right)=\{\begin{array}{c} C_{sp}{\left(\frac{\Omega }{\Omega_{0}}\right)}^{-p_{1}},\;\;\Omega \leq \Omega_{0}\\ C_{sp}{\left(\frac{\Omega }{\Omega_{0}}\right)}^{-p_{2}},\;\;\Omega \geq \Omega_{0} \end{array}$$

where Ω is the spatial circular frequency in cycle/m, $\Omega_{0}$ is the standard spatial frequency, $C_{sp}$ is the coefficient of road roughness, $p_{1}$ and $p_{2}$ are dimensionless frequency indices. $\Omega_{0}$ is the boundary between low frequencies and high frequencies in the road power spectrum. 

Further, the power spectral density is rewritten in terms of time angle frequency ω:

(6) $$S_{q}\left(\omega \right)=\frac{1}{2\pi v}S_{q}\left(\Omega \right)$$

where *v* is the speed of the vehicle.

### 3.2. Pavement Material and Motion Model

Asphalt material is widely used in the construction of road infrastructure. The layered elastic model based on the theory of multilayer linear elastic system is usually used in dynamic response analysis of asphalt pavements [20,21]. However, asphalt material is a typical viscoelastic material which has obvious stress and temperature dependence. The viscoelastic properties of asphalt material play a crucial role in pavement response and performance [22]. Therefore, linear viscoelastic material model is introduced to build pavement motion model in this paper.

**Figure 2:**
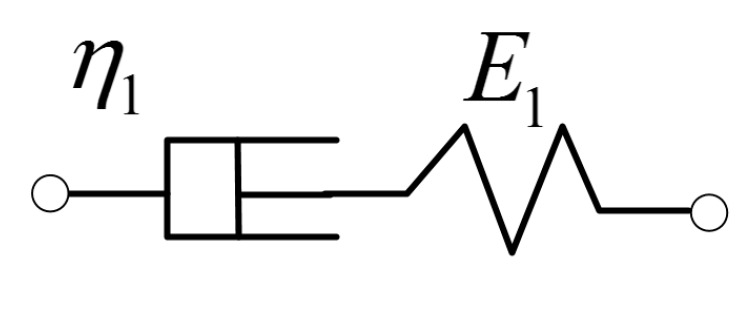
Maxwell model.

The rheological process of asphalt material is a complex process involving a variety of deformation such as elasticity, plasticity, viscoelasticity and viscoplasticity. The Maxwell model including Hooke solid and Newtonian solid (as shown in Figure 2) is a typical linear viscoelastic material model [23]. The constitutive equation and viscoelastic operator of a Maxwell model is defined as follows:

(7) $$\sigma +\frac{\eta_{1}}{E_{1}}\dot{\sigma }=\eta_{1}\dot{\varepsilon }$$

(8) $$E\left(s\right)=\frac{E_{1}\eta_{1}s}{E_{1}+\eta_{1}s}$$

where σ is stress, ε is strain, $E_{1}$ is elastic modulus, $\eta_{1}$ is viscosity coefficient. 

**Figure 3:**
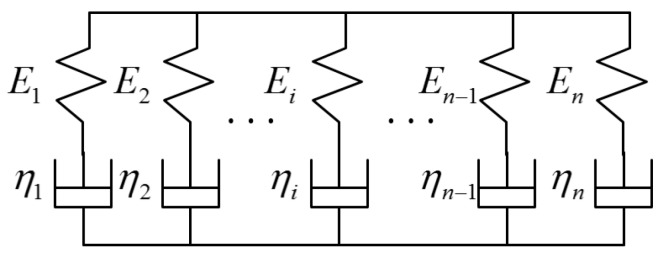
General Maxwell model.

In order to describe the complex viscoelastic properties of asphalt, a general Maxwell model (as shown in Figure 3) given in Reference [24] is used in this paper. The General Maxwell model is constructed by N Maxwell models in parallel. Thus, the constitutive equation of the General Maxwell model is formulated as:

(9) $$\overset{n}{\underset{k=0}{\sum }}p_{k}\frac{d^{k}\sigma }{dt^{k}}=\overset{n}{\underset{k=1}{\sum }}q_{k}\frac{d^{k}\varepsilon }{dt^{k}}\;$$

The viscoelastic operator of the general Maxwell model is formulated as:

(10) $$E\left(s\right)=\frac{\sum_{k=0}^{n}q_{k}s^{k}}{\sum_{k=0}^{n}p_{k}s^{k}}\;$$

where $p_{k}=A_{k}{}^{\left(i\right)}\left(k=0,1,2\ldots n\right)$, $q_{k}=B_{k}{}^{\left(i\right)}\left(k=0,1,2\ldots n\right)$. $A_{k}{}^{\left(i\right)}$ and $B_{k}{}^{\left(i\right)}$ are related to k and the detailed derivation process can be found in Reference [24]. 

Combining the general Maxwell model in Equation (9) and the theory of linear viscoelasticity material [25], the linear viscoelasticity material behavior of asphalt pavement as:

(11) $$G\left(t\right)=G_{\infty }+\overset{n}{\underset{i=1}{\sum }}G_{i}e^{-\frac{t}{\tau_{i}{}^{G}}}K\left(t\right)=K_{\infty }+\overset{n}{\underset{i=1}{\sum }}K_{i}e^{-\frac{t}{\tau_{i}{}^{K}}}$$

where G(∞),K(∞),G(i),K(i) are material constants in Pa, ${\tau_{i}}^{G}$ and ${\tau_{i}}^{K}$ are material constants in *s*. 

According to the finite element theory [25,26], the pavement motion model can be represented as:

(12) $$\left[M\right]\left\{\ddot{u}\right\}+\left[C\right]\left\{\dot{u}\right\}+\left\{R\left(u,\dot{u},\ddot{u}\right)\right\}=\left\{F\right\}$$

where [M] and [C] are the mass and damping matrices, respectively, {u} and {F} are the vectors of nodal displacements and nodal external forces respectively, {R} is the vector of the nodal internal resistance forces, which are nonlinear functions of $\left\{u\right\},\;\left\{\dot{u}\right\}$ and $\left\{\ddot{u}\right\}$. External force in {F} comes from the moving loads of vehicle.

## 4. Numerical Model of WIM System

Finite element analysis is a numerical method for the approximate solution of physics and engineering problems, which is significantly superior to theoretical analysis when studying problems with nonlinear properties and complex loads. In this section, the finite element model of a WIM system is developed. Specifically, a six-layer pavement structure model based on in the size of a single lane and two kinds of strain sensor model are constructed. A dynamic load model based on a two-axle moving vehicle is designed and implemented. The common finite element software Abaqus is used in this work. 

### 4.1. Pavement Structure Model

**Figure 4:**
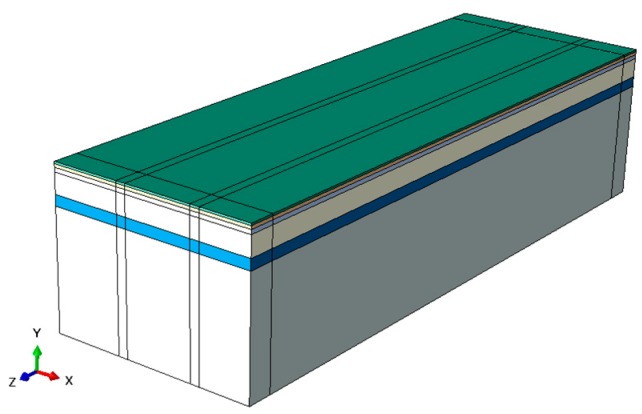
Pavement structure.

The three-dimensional structure modeling of a typical flexible pavement consists of selecting the appropriate dimensions of a finite domain and deciding the degree of its discretization. To simulate the real situation as much as possible, a single lane model is constructed, as shown in Figure 4. Specifically, the width of the lane (in *X* direction) is 4 m, the depth (in *Y* direction) is 3 m and the length (in *Z* direction) is 10 m.

For the boundary conditions, the rollers are placed on lateral planes (*ZY* and *XY* planes), which constrain the movement in *X* and *Z* directions. Moreover, the structure is constrained from displacements in all three directions in the bottom plane [18,19,20,21,22,23,24,25]. 

**Figure 5:**
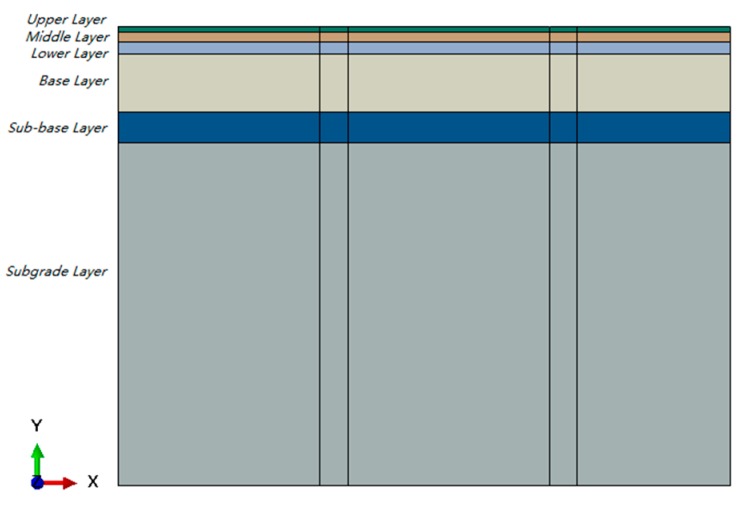
Layers of pavement.

Typical flexible pavements generally include an asphalt concrete layer (AC), a base layer (BS), a sub-base layer (SB) and a subgrade layer (SG). The subgrade layer is mainly compacted soil or clayey soil. The base layer and sub-base layer usually consist of concrete, graded broken stone, graded gravel, and so forth. Asphalt layer is of various asphalt mixture. The material and thickness of all layers are shown in Table 1. The structure of the pavement model is shown in Figure 5, in which the asphalt concrete layer is further divided into three layers: Upper layer, Middle layer and Lower layer. 

The Generalized Maxwell model in Section 3 is used to model the time-dependent shear and volumetric behavior of asphalt layer material and other layers material are elastic material. The viscoelasticity of asphalt material is defined by the Prony series of relaxation modulus in Abaqus. In our implementation, the viscoelastic parameters of the asphalt layer are based on the work [27] and the temperature is fixed at 20 °C. 

To improve the rate of convergence, the interface layers in pavement are assumed to be fully bounded, which means that the continuity conditions are satisfied at the layer interfaces and the vertical stress, shear stress and vertical displacement are the same on each side of the interface.

**Figure 6:**
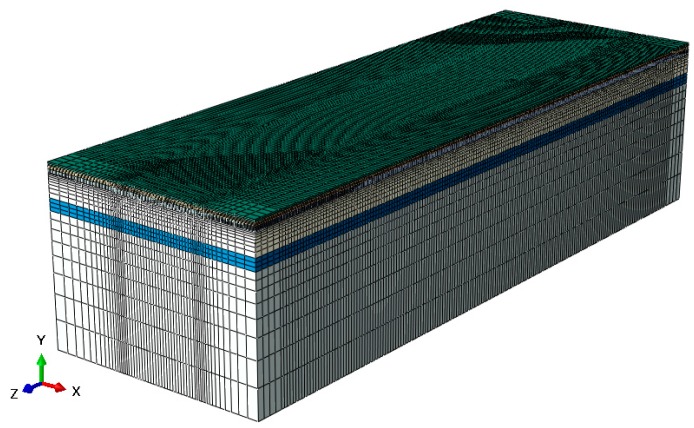
Pavement model meshed.

Taking into account both accuracy and computation cost, we divide meshes of 3D pavement model in different sizes. The meshes near the loading area and the sensors are finer allowing a higher accuracy in these regions, while the mesh become thicker as the distance gets farther from loading area. As shown in Figure 6, the pavement structure is discretized into a finite number of 8-node linear brick elements with reduced integration (SOLID C3D8R type in Abaqus program) [28]. 

### 4.2. Vehicle Load Model

Dynamic analysis technique [29] is used to properly simulate vehicle moving loads instead of static or quasi-static method. Traditional vehicle-pavement interaction analysis assumes that the tire–pavement contact stress is uniformly distributed over a circular area and equals to the tire inflation pressure. However, the real contact area is not a complete circle. Especially, in the case of wide-base tire, the contact area gets more oriented in the width and less in length comparing with that of conventional tires [30]. In our work, tire contact area is assumed to be a rectangle with a constant width. The relationship of its length and the axle load and tire pressure is formulated as follows:

(13) $$L_{1}=\frac{F_{s}}{p\cdot L_{2}}$$

where $F_{s}$ (kN) is a single static load which equals the static tire force in Equation 3, p (kPa) is the tire pressure, $L_{2}$ (m) is the width of contact area which equals the tire width and $L_{1}$ (m) is the length of contact area.

**Figure 7:**
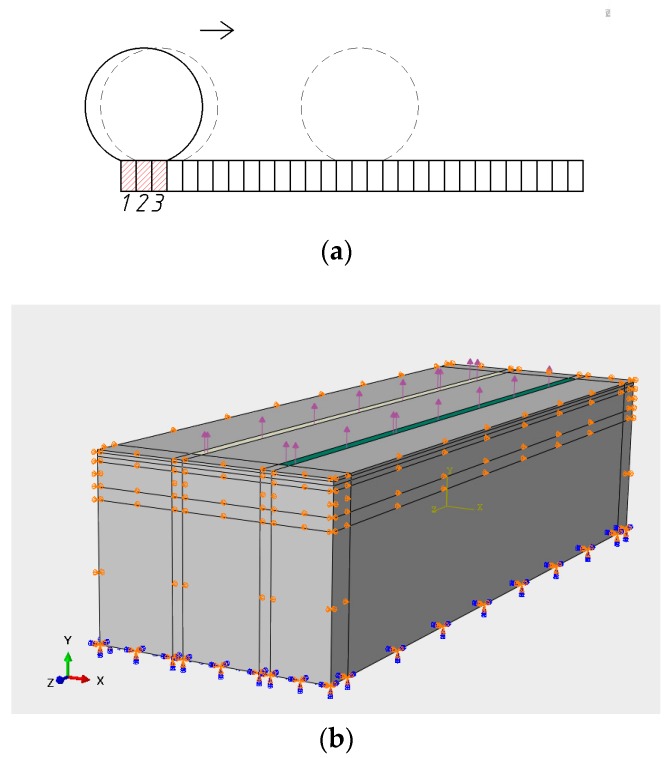
Moving load on the pavement. (**a**) Load movement method; (**b**) The position of the moving load on the pavement.

To simulate a moving wheel loading at a certain speed, the concept of continuous step loading is used. In this approach, a contact area is divided into three parts. A step loading is applied to the first part and then moves to the next in the traveling direction. When the step loading reaches the last part, a single wheel path is completed. Figure 7 shows the concept of step loading. Specifically, each part is a rectangle in our simulation, which is $L_{1}/3$ in length and $L_{2}$ in width. The time period is calculated by $L_{1}/\left(3\times v\right)$, v is the speed of the vehicle.

In our work, road grade is assumed to be A. According to ISO/TC108 standard, the parameters in the road roughness model are as follows: $\Omega_{0}=1/2\pi \;\mathrm{cycle}/m$, $C_{sp}=8{\mathrm{cm}}^{3}$. The road roughness in time domain is generated by filtering Gaussian white noise method [31]. Dynamic tire force in the time domain is calculated by a quarter-vehicle model implemented in MATLAB according to the input of road roughness [19,32,33,34].

In our implementation, the common vehicle parameters are set as follows: the wheelbase is 3 m, the wheel center distance is 1.5 m. Furthermore, six vehicle load models with different static wheel loads and speeds are simulated. The parameters of each model are presented in Table 2.

### 4.3. Sensor Model

The deformation of pavements under load causes the deformation of sensor external structure, which further leads to changes in sensor output. There are many kinds of embedded strain sensors, such as asphalt strain gauges [4] and fiber optic strain sensor [35]. In this paper, a sensor structure model is simplified to a cylinder because most of the asphalt strain sensors are of this shape. Moreover, the values of sensor strain are used as sensor signal outputs since the relationship between them is supposed to be linear. 

**Figure 8:**
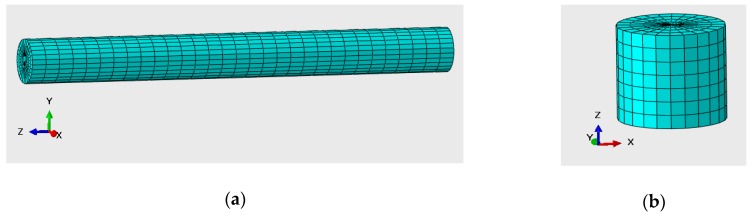
(**a**) Horizontal strain sensor; (**b**) Vertical strain sensor.

There are two kinds of sensors widely used in embedded WIM system [12,36,37]: horizontal strain sensor and vertical strain sensor. A horizontal strain sensor has to be installed in parallel to the road surface in order to measure the horizontal strain, while a vertical strain sensor is perpendicular to the road surface for sensing vertical strain. Figure 8 demonstrates these two kinds of sensor models.

**Figure 9:**
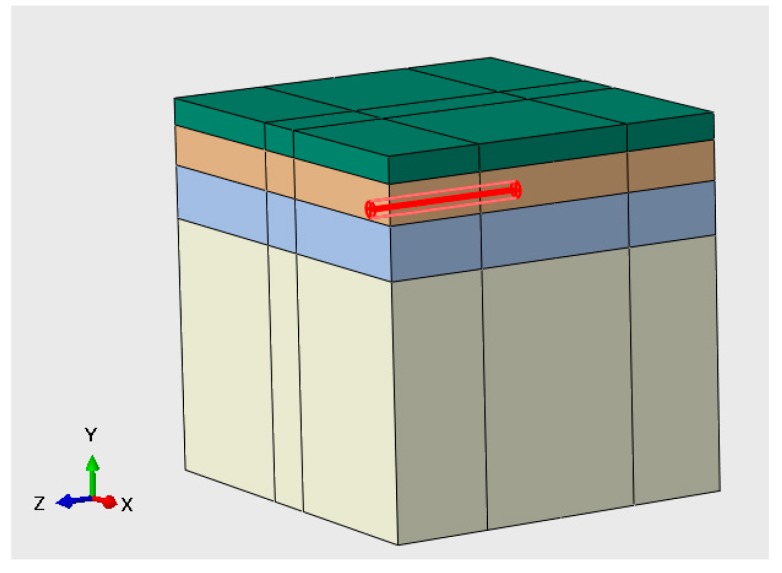
Sub-model with a horizontal strain sensor. The red is a horizontal strain sensor embedded in the lower layer.

It would be hard to balance accuracy and computation speed when the lengths of the pavement model and the sensor model vary greatly. To solve this issue, the sub-model method in Abaqus is used. First the global model containing the whole pavement and sensors is analyzed with a relatively coarse mesh. When the results from the global model are satisfactory, a sub-model including a sensor and a part of the pavement with finer meshes is set up. Note that the sub-model is located at the same position as the global model because the results from the global model will be used to apply boundary conditions to the sub-model. Finally, the sub-model is used for analysis and gets more accurate results. The sub-model is shown in Figure 9.

## 5. Simulation and Analysis

**Figure 10:**
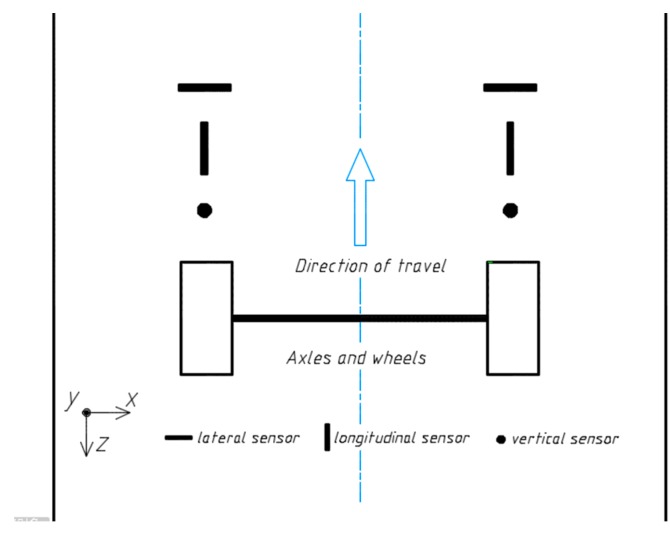
Top view of pavement, vehicles and sensors.

Besides the dynamics of vehicles, many factors could affect a WIM system performance, such as sensor intrinsic error and weather condition. In this work, we focus only on the impact of sensor layout on the measurement of dynamic load. As shown in Figure 10, the coordinate axes used in simulation are defined as follows: the direction of travel (or longitudinal in brief) is taken as the positive z-axis while the y axis is normal to the ground and positive downward. Horizontal strain sensors could be broken down into two types according to their installation methods: longitudinal and lateral. Vertical strain sensors have to be embedded perpendicularly to the ground.

**Figure 11:**
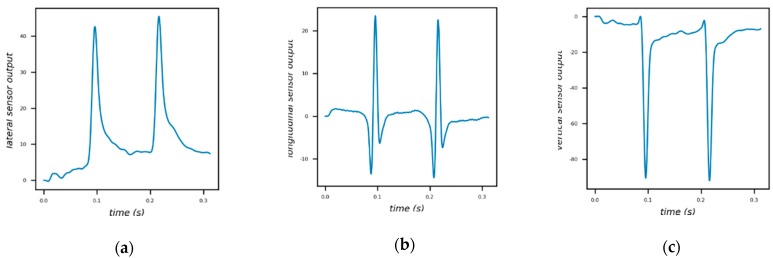
(**a**) Lateral sensor; (**b**) Longitudinal sensor; (**c**) Vertical sensor.

In order to reduce the time cost during simulation, there are only 122 incremental steps in the analysis of the model, resulting in less sensor data. To alleviate this problem, B-spline interpolation is used to supplement data and make curves smoother [3,4,12]. But even so, a simulation process of our model takes about 8 hours on a workstation (Intel Core i7-4940MX, 32GB memory, NVIDIA Quadro K2100M. Lenovo, Beijing, China.). Figure 11 depicts the outputs of lateral sensor, longitudinal sensor and vertical sensor under a moving two-axle vehicle. The signal peak in the outputs indicates the strain response measured when an axle passes the sensor. It is worth noting that the strain values generated by simulation are linearly scaled in terms of signal amplitude for easy viewing.

To evaluate the accuracy of estimation, the weighing error is defined as the difference between the static weight and the measured weight by a sensor:

(14) $$A=\frac{\left|W_{m}-W_{s}\right|}{W_{s}}\times 100\%$$

where A is relative error, $W_{m}$ is wheel weight estimated by the sensor, $W_{s}$ is static wheel weight. 

The dynamic force applied to the ground by a moving wheel varies due to the combined contribution of vehicle oscillation and road roughness. Therefore, multiple sampling points along the travelling direction are used in multi sensor WIM systems in order to improve the accuracy of estimation. In order to reduce the impact of dynamic response, each experiment in Table 2 was repeated ten times under different random dynamic loads generated by Section 4.2. Finally, the wheel weight estimated $W_{m}$ is the average of the peak values of the sensor outputs from ten loadings [3]. 

### 5.1. The Impact of Installation Depth

**Figure 12:**
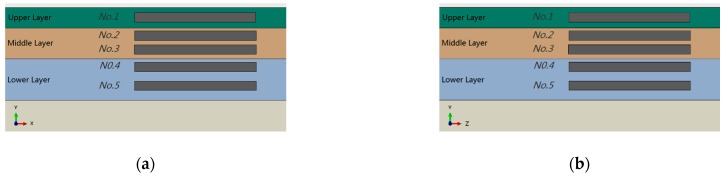
(**a**) Lateral sensor placement; (**b**) Longitudinal sensor placement.

**Figure 13:**
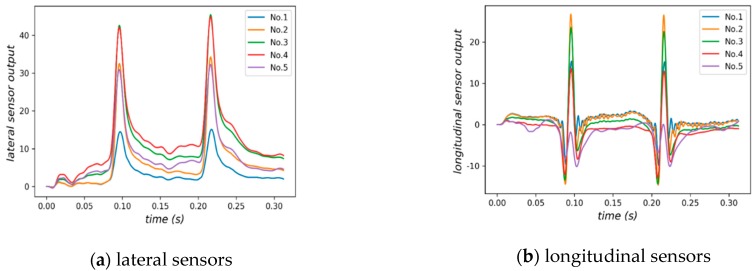
Outputs of sensors placed in different depth with NO. 1 vehicle load model.

The mechanical behavior of pavements under moving vehicles is crucial for pavement design and evaluation. A large number of different models have been proposed in order to characterize the dynamic response of pavements to moving loads [38]. Here, we focus on the impact of sensor placement on accurately capturing dynamic load induced in the pavement by moving vehicles. As shown in Figure 12, we placed five lateral sensors and longitudinal sensors in different asphalt layers to identify strain effect on sensors. The output signals of these sensors under NO. 1 vehicle load model in Table 2 are given in Figure 13. It can be seen from Figure 13 that the output signals have the similar waveforms but differ greatly in terms of peaks. The sensor outputs with other vehicle load models are similar to that of model NO. 1.

**Figure 14:**
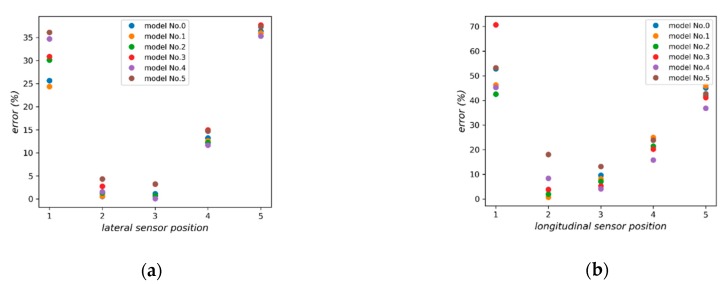
(**a**) Relative errors of lateral sensors in different depth, (**b**) Relative errors of longitudinal sensors in different depth.

Furthermore, the relative errors of the sensors are calculated according to Equation (14). As shown in Figure 14a, for the lateral sensor array, NO. 2 and 3 sensors perform best and NO. 1 and 4 sensors perform the worst. The output of NO. 1 sensor behaves unstably in both situations. Figure 14b shows a similar result, indicating that longitudinal sensors in the middle layer perform better.

**Figure 15:**
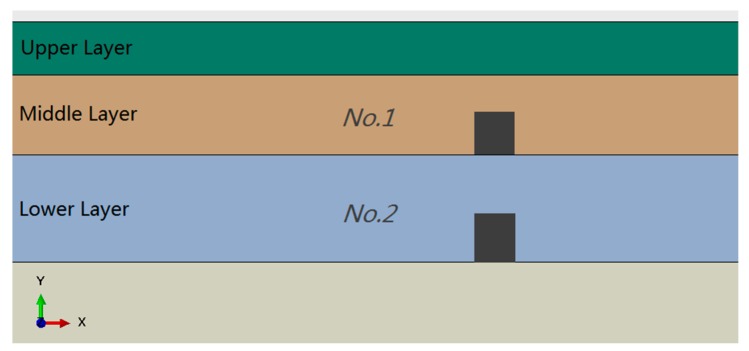
Placement of vertical sensors.

**Figure 16:**
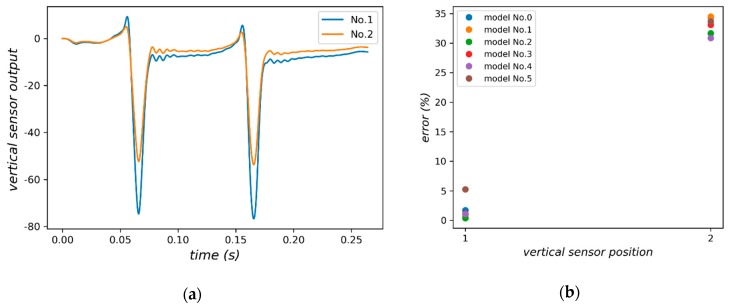
(**a**) Signal of vertical sensor located in different depth; (**b**) Relative errors of vertical sensors in different depth.

For vertical sensors, as shown in Figure 15, only two sensors could be placed in the middle and lower layer respectively limited by the size of sensors. As shown in Figure 16, the output of sensors has a regular waveform, that is, a weak positive signal and a large negative signal appear before and when the wheel reaches the sensor. It can be seen from Figure 16b that NO. 1 sensor performs better than NO. 2 sensor under all vehicle load models.

Due to the complexity of interaction between road and vehicle, quantifying the relationship between the depth of a sensor and its outputs under various conditions remains a challenge. The results of our simulation suggest that the depth of sensors embedded and the road material should be considered in order to improve the ability of dynamic response sensing in the design of a WIM system. 

### 5.2. The Impact of Distance between Tires and Sensors

As mentioned in Section 2, a number of multi-sensor WIM systems with different sensor layouts have been developed. These systems give promising results under ideal conditions, that is, the wheels of the passing vehicle move directly above the embedded sensors. However, we cannot guarantee that the vehicle will travel under the ideal trajectory in practice. The effects of distance between tires and sensors should be considered in the placement of sensors. 

**Figure 17:**
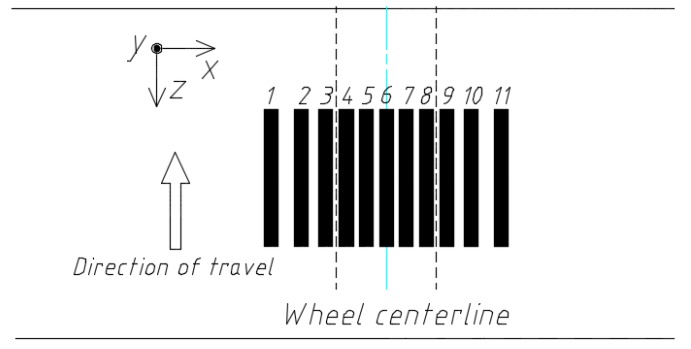
Placement of longitudinal sensors.

**Figure 18:**
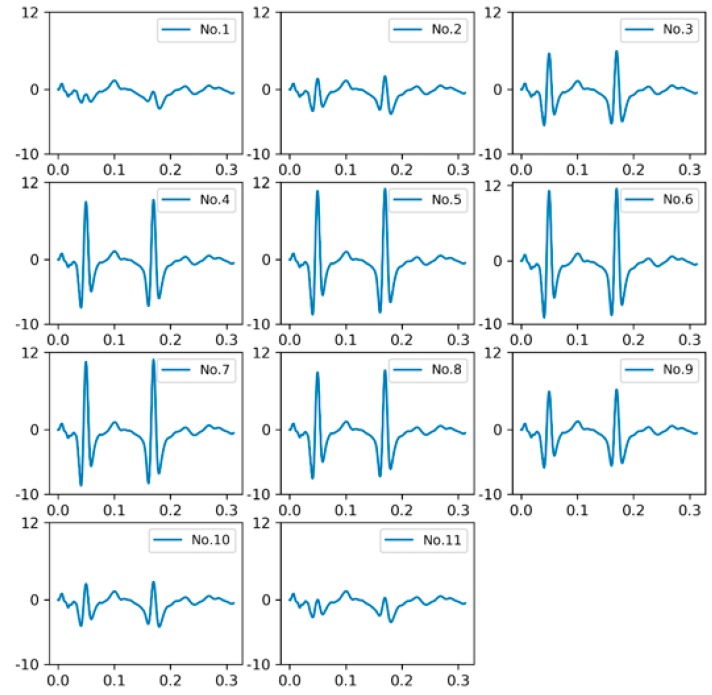
Outputs of longitudinal sensors.

As shown in Figure 17, we placed 11 longitudinal sensors along z direction (perpendicular to the traveling direction), in which the lateral offset of each sensor from the centerline of the wheel is −210, −155, −101, −73, −36, 0, 36, 73, 101, 155, 210 (mm), respectively. The area between the two dotted lines represents the trajectory of a wheel. Figure 18 shows the output of each sensor under the vehicle load model NO. 1.

**Figure 19:**
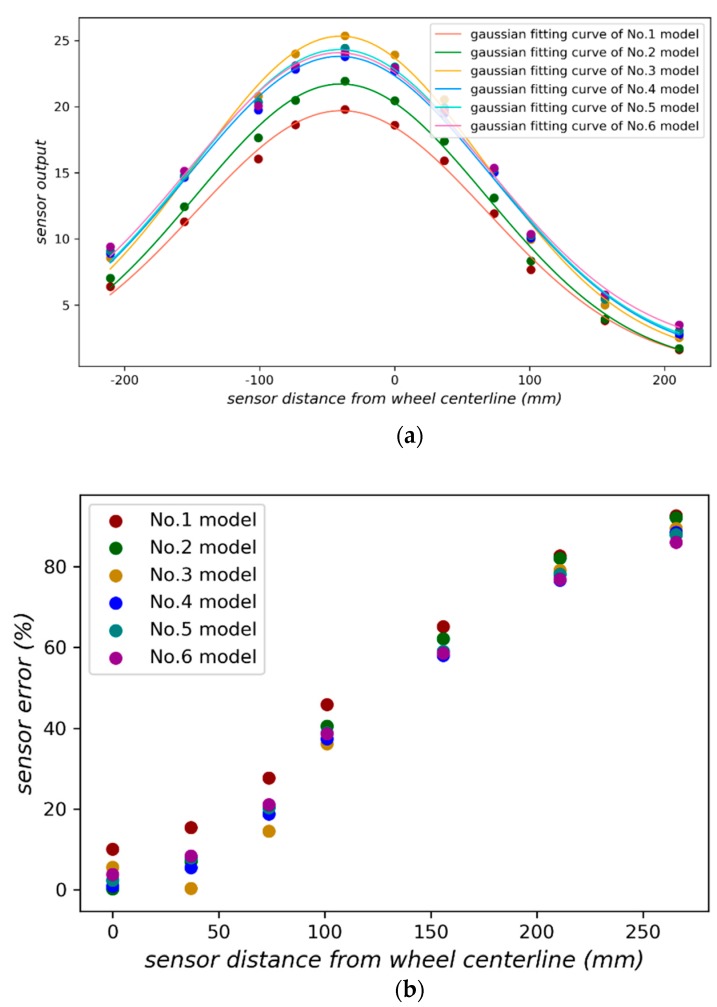
(**a**) Gaussian fitting curves for the placement in Figure 17; (**b**) Sensor position offset and weighing error.

When a wheel passes over the sensors, the closer the sensor is to the centerline of the wheel, the stronger the output signal is. The wheel weight measured and lateral offset of sensor are approximately Gaussian [4]:

(15) $$W_{m}=ae^{-\frac{1}{2}{\left(\frac{x-b}{c}\right)}^{2}}+d$$

where $W_{m}$ is the wheel weight measured by the sensor, x is lateral offset between the sensor location and centerline of the wheel. Figure 19a shows the Gaussian curve fitted with six vehicle load models in Table 2. The weighing error of each sensor is depicted in Figure 19b. Once the sensor deviates far from the wheel, the weighing error will increase rapidly. 

**Figure 20:**
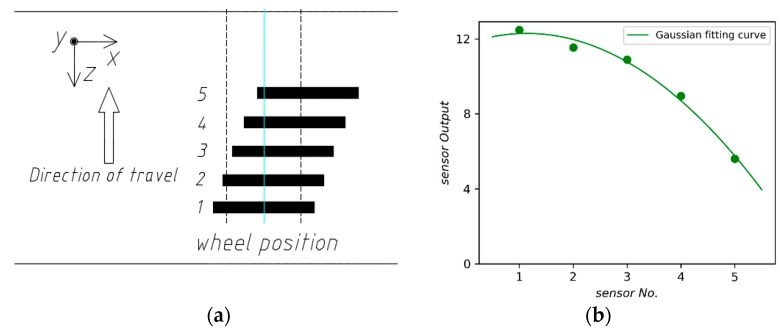
(**a**) The array with five lateral sensors; (**b**) Gaussian fitting curve.

Accordingly, a sensor array consisting with lateral sensors (Figure 20a) is constructed and the output of each sensor is shown in Figure 20b. Consistent with longitudinal sensors, the weighing error increases with the lateral offset of a sensor. 

**Figure 21:**
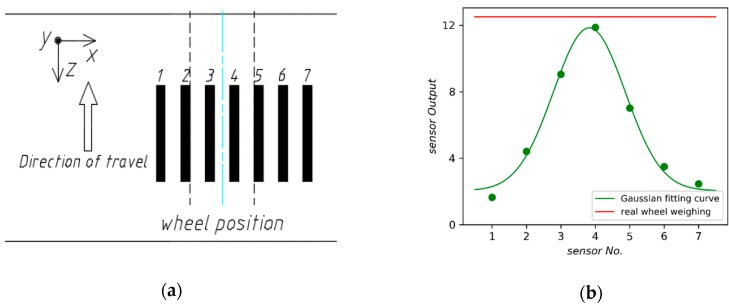
(**a**) The array with seven longitudinal sensors; (**b**) Gaussian fitting curve.

**Figure 22:**
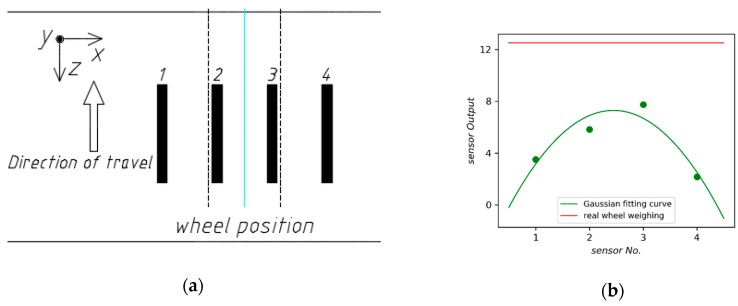
(**a**) The array with four longitudinal sensors; (**b**) Gaussian fitting curve.

Consequently, it is to be expected that more sensors should be deployed to improve the accuracy of WIM systems. However, it is unpractical from an economic point of view. To solve the problem, the method of Gaussian curve fitting has been proposed [4]. We evaluate the method by two layouts of sensors. Figure 21 presents an array with seven sensors evenly spaced and its Gaussian fitting curve provided by the least square method. However, the dynamic force applied by moving vehicle would decay rapidly due to the mechanical properties of the pavement. As shown in Figure 22, the weighing error is too large to be used when the number of sensors in the array drops to four. 

## 6. Conclusions

The multiple-sensor WIM system utilizes an array of sensors embedded under the pavement to detect the static weight of vehicles while in motion. As a part of a WIM system, the pavement will inevitably influence the performance of the system. In this paper, the finite element model of a WIM system was developed, which allows sensors to be installed anywhere in the pavement model. The numerical simulation was performed to analyze the effects of sensor location on the ability of dynamic load sensing. In our simulation setting, the sensors placed in the middle layer of asphalt concrete layer have better sensing ability for dynamic responses. In addition, our results validate that dynamic response of the pavement fits a Gaussian distribution centered at the wheel position. Gaussian curve fitting could be used to reduce estimation error. However, it would fail when the space between the sensors is too large.

The quarter vehicle model used in this paper only considers single wheel load. Extending the vehicle model to enable multiple wheel loadings is a topic for future work, which will allow us to measure the impact of the entire vehicle load on sensor placement. In another aspect, the pavement model used in this paper is an idealized model without considering pavement damage and temperature change. In addition, our approach does not take the structural characteristics of bridges into consideration in modeling of road surface. On the basis of the potential applications of our WIM model, it may be concluded that these problems are worthy of further study.

## Figures and Tables

**Table 1:** Parameters of each layer.

Structure Layer	Material	Code Number	Thickness/cm	Elastic Modulus/Mpa	Poisson Ratio	Mass Density/(kg/m^3^)
Upper layer	Basalt fiber asphalt mixture	AC-13C	4	--	0.3	2300
Middle layer	Modified asphalt mixture	Sup-20	6			
Lower layer	Heavy traffic asphalt mixture	Sup-25	8			
Base layer	Cement stabilized macadam	CSM	38	1500	0.2	2300
Sub-base layer	Graded broken stone	GM	20	450	0.3	2100
subgrade layer	Compacted soil	SG	224	45	0.4	1850

**Table 2:** Parameters of Vehicle load models.

Number	Speed (m/s)	Static Wheel Load (kN)	Tire Pressure (MPa)	Length of Contact Area (mm)	Tire Width (mm)
1	25	25	0.8113	192	184
2	25	30	0.8739	231	184
3	25	35	0.9365	270	184
4	30	25	0.8113	192	184
5	30	30	0.8739	231	184
6	30	35	0.9365	270	184
